# Gauging the Acceptance of Telemedicine in Postoperative Evaluation of Uncomplicated Laparoscopic Appendectomy and Cholecystectomy

**DOI:** 10.1089/tmr.2023.0027

**Published:** 2023-08-24

**Authors:** Lily Choi, Courtney Riedinger, Kent Gardner, Craig Ziegler, Reginald Brinson, Erica Sutton

**Affiliations:** ^1^ University of the Incarnate Word School of Osteopathic Medicine, San Antonio, Texas, USA.; ^2^ Department of Obstetrics and Gynecology, The Ohio State University College of Medicine, Columbus, Ohio, USA.; ^3^ Office of Undergraduate Medical Education, University of Louisville School of Medicine, Louisville, Kentucky, USA.; ^4^ Office of Information Technology, Morehouse School of Medicine, Atlanta, Georgia, USA.; ^5^ Department of Surgery, Morehouse School of Medicine, Atlanta, Georgia, USA.

**Keywords:** general surgery, telemedicine, telehealth, telecommunications

## Abstract

**Background:**

Telemedicine is a rising field, with continuous expansion into different realms of health care delivery. However, minimal research has been done to analyze the utilization in surgical specialties. This study aims to assess satisfaction and acceptance of postoperative telehealth care after uncomplicated general surgery cases.

**Methods:**

Patients who had undergone uncomplicated laparoscopic cholecystectomy or uncomplicated laparoscopic appendectomy were eligible to be enrolled in this study. Patients with gangrenous gallbladder, malignancy, operative complications, or appendix perforation were excluded. The experimental group underwent postoperative follow-up within a web-based platform (http://bluejeans.com), whereas the control group had an in-person clinic visit. Survey results containing satisfaction, comfort, and time usage were obtained. Likert scale 1–5 was utilized to quantify responses.

**Results:**

Thirty patients were enrolled into this prospective single intervention trial (20 experimental, 10 control). Ninety percent (*n* = 18) of the experimental group stated satisfaction with their visit, and 75% (*n* = 15) would suggest telemedicine usage to other physicians. Postoperative visit satisfaction was not statistically different between the experimental and control groups (4.2 vs. 4.5, *p* = 0.124). A higher percentage of the control group took >3 h for the visit than the telemedicine group (30% vs. 15%), with two individuals in the control group dedicating their full day to the visit, compared with zero individuals in the experimental group. Comfort with technology used during the visit was not statistically different between the telemedicine and in-person groups (4.35 vs. 4.5, *p* = 0.641).

**Conclusions:**

Telemedicine for postoperative evaluation on selective general surgery cases is feasible and provides adequate patient satisfaction and improved time utilization.

## Introduction

The 2020 SARS-CoV-2 pandemic necessitated the rapid and widespread use of telemedicine for health care delivery in the United States and worldwide.^1^ Before this pandemic, telemedicine was increasingly being used to improve accessibility and efficiency of health care delivery, although at a less fervent pace. Although the need to connect patients and health care providers has pushed all into heretofore unfamiliar virtual environments, little is known about the efficacy and patient acceptance of these interactions for highly tactile specialties such as general surgery.^2^

We performed a pilot study employing telemedicine for emergency general surgery patients who underwent laparoscopic appendectomy or laparoscopic cholecystectomy for uncomplicated acute appendicitis or cholecystitis to assess feasibility and patient acceptance of telemedicine for routine postoperative evaluation.

The utilization of telemedicine has been limited in surgical specialties, with increasing research in recent years with the expansion of e-health-based health care^3^ Studies that have been performed in the surgical setting have shown increased benefits to patient care with improvements in monetary savings as well as decreasing traveling distances to seek further care.^3–7^ Specifically within general surgery, decreases within travel time were seen as well as increases in clinic availability with the transition to an online care delivery model.^8,9^ Few complications associated with telemedicine-based postoperative surgical care were seen throughout studies of multiple surgical specialties.^10–12^

In 2020, the SARS-CoV-2 pandemic produced a rapid expansion of the services and terms of distanced health care, with new challenges associated with the limitation of face-to-face consultation and patient interaction.^13^ Chao et al. investigated the surgical conversion rates per surgical specialty during the COVID-19 pandemic. The peak mean of telehealth conversion for general surgery was 4.9% compared with that for urology and neurosurgery whose peak mean conversion rates were 14.3% and 13.8%, respectively.^14^ With former research showing the possible monetary and time benefits to telehealth utilization, its potential and patient acceptance within general surgery is yet to be fully explored.

The term “telemedicine” has come to refer to a wide array of health-related services in the past 3 years. Former studies have assessed quantitative efficiency of an e-health care model. However, although many of these studies focused on traveling distance and time, subjective patient satisfactions in routine general surgery cases have not been extensively documented. In a time when the incorporation of technology into health care is being more frequently utilized, it is crucial to understand patient acceptance to this newer medium of patient care. The aim of this study is to assess patient acceptance to postoperative telehealth care within general surgery. Our hypothesis was that telemedicine would provide adequate follow-up care and be favorably received by such patients.

## Methods

Patients were eligible for the study if they were between 18 and 90 years old and had undergone an uncomplicated laparoscopic appendectomy or cholecystectomy. Patients were considered to have a complicated appendectomy and ineligible to participate if they had evidence of perforation of the appendix or malignancy discovered by pathological examination of the specimen. Patients were considered to have a complicated cholecystectomy and ineligible to participate if they were discovered to have a gangrenous gallbladder, suspected of any hepatobiliary malignancy, or any injury to the biliary tree or hepatic vasculature. All patients underwent surgery by a single surgeon who completed a fellowship in laparoscopic surgery.

To participate in the study, it was necessary for patients to have access to visual communication technology. If patients did not have access, a webcam was offered to the patient at no charge. The experimental group was set at 20 and the control group set at 10. Informed consent was obtained in person after surgery at the index hospitalization or through telephone within 2 weeks of surgery. After informed consent, the patients in the experimental group were given a link to a secure and private web-based meeting platform (http://bluejeans.com).

Instructions were also given over the phone about how to use a smartphone or computer with a webcam to log in at their specified meeting time with a meeting ID and password. Patients were allowed to select the location of their telemedicine follow-up visit and notified that they would be able to see and speak to their physician in a manner similar to an in-office interaction.

**FIG. 1.**
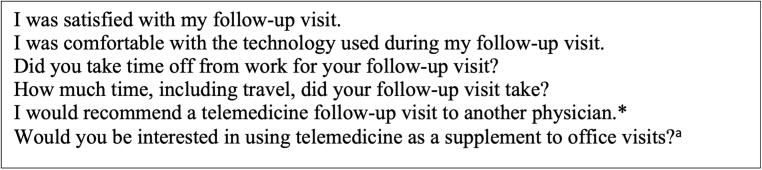
Online survey questions given to patients in both the experimental group and the control group after postoperative follow-up visit. *Question given only to experimental group. ^a^Question given only to control group.

After the telemedicine follow-up was completed by the patient and surgeon, visits were evaluated with an online survey. Primary outcomes measured included patient satisfaction with follow-up visit, time off from work and travel time that was required for the visit, patient level of comfort with the technology used, and the patient's receptiveness to future telemedicine visits (Likert scale 1–5 with 1 being strongly disagree, 5 being strongly agree) (Fig. 1). The questionnaire results from this group were then compared with those from a control group of patients who experienced a traditional in-office follow-up visit.

Descriptive statistics such as mean, standard deviations, frequencies, and percentages were calculated and presented for the telemedicine and control groups. The telemedicine group and the control group were compared using the independent samples *t*-test, the Mann–Whitney *U*, and Pearson chi-square statistics. Statistical significance was set at convention as *p* < 0.05. SPSS version 21.0 was used to analyze the data.

## Results

**FIG. 2.**
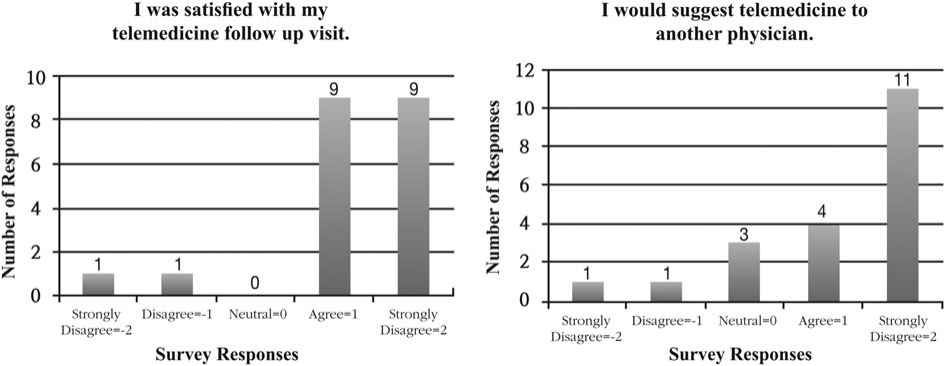
Likert scale (1–5) scoring results for patient satisfaction survey questions given to experimental telemedicine group (*n* = 20).

Sixty-three patients were approached about participation, 20 of whom entered the study as telemedicine participants (31.7%). The 43 patients who did not participate in the study were invited to serve as controls, 10 of whom entered the study (23.3%). Of the telemedicine participants, 90% were satisfied with their telemedicine visit, and 75% would suggest the use of telemedicine to another physician (Fig. 2). Satisfaction with the postoperative visit was not significantly different between the telemedicine group and control group, 4.2 versus 4.5 (*p* = 0.124) (Table 1).

**Table 1.** Survey Question Responses for Telemedicine Patients and Controls

			Strongly disagree/disagree (1–2)		Neutral (3)		Agree/strongly agree (4–5)				
		*n*	Frequency	%	Frequency	%	Frequency	%	Mean	SD	*p*
I was satisfied with my follow-up visit	Telemedicine	20	2	10	0	0	18	90	4.20	1.06	0.124
	Nontelemedicine	10	1	10	0	0	9	90	4.50	1.27	
I was comfortable with the technology used during my follow-up visit	Telemedicine	20	2	10	1	5	17	85	4.35	1.27	0.641
	Nontelemedicine	10	1	10	0	0	9	90	4.50	1.27	
			**Yes**	**%**	**No**	**%**					
Did you take time off from work for your follow-up visit?	Telemedicine	20	1	5	19	95					0.031
	Nontelemedicine	10	4	40	6	60					

SD, standard deviation.

Only 1 telemedicine study participant took time off from work and 17 participants (85%) reported that their telemedicine visit took 3 h or less of their time in total. In comparison, 30% of controls took >3 h including travel time to complete their in-office follow-up visit, with two participants (20%) devoting their full day to the visit (Table 2).

**Table 2.** Survey Question Response: How Much Time Did You Spend on Your Follow-Up Visit?

		<1 h		1–2 h		2–3 h		4 h		>5 h		Full day	
	***n***	Frequency	%	Frequency	%	Frequency	%	Frequency	%	Frequency	%	Frequency	%
Telemedicine	20	2	10	8	40	7	35	2	10	1	5	0	0
Nontelemedicine	10	0	0	7	70	0	0	0	0	1	10	2	20

A final consideration when using telemedicine is the comfort level with the technology used. Eighteen out of our 20 study participants (90%) were comfortable with the technology they were required to use to operate the web-based meeting platform, similar to controls who encountered the routine use of technology in the office, such as check in software and messaging systems (4.35 vs. 4.5, *p* = 0.64) (Table 1). Finally, 70% of the control arm reported that they would be interested in using telemedicine as a supplement to office visits (Table 3).

**Table 3.** Survey Question Responses Specific for Telemedicine Patients and Controls

		***n***	Strongly disagree/disagree (1–2)		Neutral (3)		Agree/strongly agree (4–5)			
			Frequency	%	Frequency	%	Frequency	%	Mean	SD
I would recommend a telemedicine follow-up visit to another physician	Telemedicine	20	2	10	3	15	15	75	4.15	1.18
Would you be interested in using telemedicine as a supplement to office visits?	Nontelemedicine	10	7	70	3	30				

## Discussion

Telemedicine and teleconsultation have shown immense benefit in many fields of medicine, but limited research has been done on its application to general surgery, and less so to emergent surgical cases.^2^ Utilization in a highly tactile specialty such as surgery remains unclear with the lack of expansive research. This study aimed to highlight patient acceptance to postoperative telehealth care within general surgery. We assessed patient satisfaction and acceptance regarding the usage of telemedicine for postoperative follow-up for uncomplicated laparoscopic cholecystectomies and uncomplicated laparoscopic appendectomies. Our study established that telehealth postoperative visits are a satisfactory and adequate option for patients, with minimization of interruption to their daily life schedule.

Patient satisfaction showed promising results with 90% of telemedicine patients reporting being satisfied with their visit, as well as 75% of patients stating that they would recommend the usage of telemedicine to another provider. With no statistically significant difference in satisfaction between the telemedicine group and the control group, we can determine that patients are able to receive adequate and satisfactory care without the difficulties associated with allotting time to visit their provider in person.

In a review of postdischarge surgical care, Gunter et al. state that round-trip mileage for appointments ranged from 128.1 km to 590.9 km, with travel time ranging from 77.5 to 317 min.^3^ This alongside the time dedicated to find childcare and find coverage for work may make access to care and follow-up difficult for many patients. Postoperative follow-up from an electronic source minimizes need for obstacles that would require time and resource consumption, providing a faster and more easily accessible option that minimizes inconveniences in life.

With the advancement of technology, systems have offered more efficient capacities to practice medicine, allotting for complexities of life.^15^ One of the benefits of telemedicine most frequently noted by our patients was that they were not required to take off work for these visits, with an 87.5% reduction in patients taking time off work from the control group to the telemedicine group. Taking time off from work or school may not be a feasible option for many, and telemedicine offers an alternative solution to these challenges for patients.

We found statistically significant results indicating that those who participated in a telemedicine visit during this study indicated a positive impact on their need to take time off from work to facilitate their follow-up visit. This may especially benefit lower socioeconomic status individuals. Monetary constraints associated with decreased time or ability for work, finding childcare, and transportation difficulties may all prove obstacles to care and follow-up with lasting impacts on patient life and livelihood. A review of surgical telemedicine postoperative care found that telemedicine visits lead to an average savings of $176.^3^ Telemedicine could be an important step in the right direction for overcoming current issues with patient access to care and increasing health care costs.

A concern with this study was the familiarity and ability to utilize a digital interface for these visits, with the potential differences of comfort level of technology between patients. Notably, 43 patients declined to participate in the study group, a possible source of selection bias in favor of telemedicine. With coaching, patients were able to log on and attend the visits in our study with little difficulty, with no statistically significant difference in comfort level of technology used in the telemedicine group versus the control group. Further research with larger sample size of age range may benefit confirmation of ability and widespread inclusivity of telemedicine follow-up.

Our study looked at uncomplicated laparoscopic cholecystectomies as well as uncomplicated laparoscopic appendectomies. Additional research with inclusion of more complex general surgery cases and their perioperative management through telehealth services would prove beneficial to demonstrate the potential as well as the limitations to telemedicine care in surgical settings.

Application to rural settings may reveal further benefit of the usage of telemedicine due to increasing specialist accessibility to areas of general surgeon need. In rural areas, access to a general surgeon may require that patients travel 2–3 h for emergency general surgery care. Further studies could be done to target satisfaction and receptivity of telehealth services to rural areas. If acceptable to patients, telemedicine could reduce geographic disparities in access to surgeons seen by rural Americans.

Before the SARS-COV 2 pandemic, Nikolian et al. looked at patient outcomes and satisfaction rates of postoperative follow-up in the acute care surgery service.^16^ This study found no difference in postoperative complications in 233 patients who had undergone a videoconference call to perform follow-up care and assessment. High (77–94%) satisfaction rates were present in the 34 patients who were polled. Daily life interruptions such as having to take off work were not measured in this study.

Cremades et al. also assessed for satisfaction and postoperative outcomes in patients enrolled from 2017 to 2018 and presented similar findings with no differences in patient satisfaction and clinical results of the 100 patients who underwent videoconference call follow-up.^17^ This study was completed in Spain and assessed a numerical value of general satisfaction rather than obtaining a broader patient survey.

Although the role telemedicine will play in mainstream health care remains uncertain, these results along with our findings may indicate that patients benefit from telemedicine. Data continually show improved access to medical services and care delivery without sacrifice of satisfaction. The potential benefits of telemedicine to both patient and physician demonstrate that further research is necessary into additional avenues in which telemedicine can be used, especially after its expansion in 2020.

We believe that the satisfaction and acceptance seen in our patients are promising in an era wherein the ever-growing demand for specialty medical care outpaces the supply of medical professionals. General surgery workforce shortages have increased and are expected to be at up to 21% by 2050.^18^ As patients become more accepting of an online option to contact their medical provider, care will be made more accessible through mediums such as live video visits, phone visits, and electronic messaging usage. This would also benefit in opening overall clinic availability as well as availability for patients who may not be able to utilize an online interface or patients who would benefit from seeing their surgeon in person.

Overall, further research would expand the feasibility of telemedicine in tactile specialties such as general surgery. Studies that portray more complex cases, geographical differences, and socioeconomic boundaries may showcase the exact benefit and acceptance of telehealth services across a multitude of populations. However, with the progressive utilization of technology in health care, the field of telemedicine has only grown and proven benefit in multiple settings. It would only be natural for us to strive to find modern methods and solutions to continue to improve the delivery of health care in surgical settings.

## Limitations

Limitations were present within our study. This study contained a small sample size, which may have skewed results and may not be an accurate representation of the population. A further study with an increase in sample size and pool may provide more conclusive data. Second, this study was done with only uncomplicated cases dealing with two distinct pathologies. Although we believe that telemedicine may provide benefit within a wider range of surgical cases, further expansion of data may be needed. Third, the surgeries in this study were performed by a single surgeon, which could have introduced bias. Future studies would benefit from the data from cases from multiple surgeons.

Fourth, we understand that selection bias favoring the telemedicine group may have been introduced. This study involved voluntary participation for inclusion into the experimental group, with 43 individuals declining participation. With possible selection bias, satisfaction with postoperative telemedicine usage could have been magnified. Finally, this study contained lack of longitudinal follow-up. These surveys only contained patient satisfaction pertaining to initial follow-up visit, but long-term satisfaction was not assessed. This may have inflated a favorable initial result, and further studies would benefit from longitudinal data measuring patient satisfaction.

## Conclusions

The American Telemedicine Association defines telemedicine as the use of medical information exchanged from one site to another through electronic communications to improve a patient's clinical health status.^19^ Telemedicine use has increased exponentially as a result of improved and cost-effective technology. This study suggests that it is feasible to use telemedicine in the follow-up of patients who have had an uncomplicated laparoscopic appendectomy or uncomplicated laparoscopic cholecystectomy. Patient satisfaction is reasonably high with telemedicine in the postoperative setting. Future studies should be conducted examining the use of telemedicine for more complex perioperative care of the surgical patient.

## Abbreviation Used

SD: standard deviation

## References

[1] Hollander JE, Carr BG. Virtually perfect? Telemedicine for Covid-19. N Engl J Med 2020;382(18):1679–1681. 32160451 10.1056/NEJMp2003539

[2] Demartines N, Freiermuth O, Mutter D, et al. Knowledge and acceptance of telemedicine in surgery: A survey. J Telemed Telecare 2000;6(3):125–131. 10912328 10.1258/1357633001935167

[3] Gunter RL, Chouinard S, Fernandes-Taylor S, et al. Current use of telemedicine for post-discharge surgical care: A systematic review. J Am Coll Surg 2016;222(5):915–927. 27016900 10.1016/j.jamcollsurg.2016.01.062 PMC5660861

[4] Cleeland CS, Wang XS, Shi Q, et al. Automated symptom alerts reduce postoperative symptom severity after cancer surgery: A randomized controlled clinical trial. JCO 2011;29(8):994–1000. 10.1200/JCO.2010.29.8315 PMC3068055 21282546

[5] Canon S, Shera A, Patel A, et al. A pilot study of telemedicine for post-operative urological care in children. J Telemed Telecare 2014;20(8):427–430. 25316038 10.1177/1357633X14555610

[6] Viers BR, Lightner DJ, Rivera ME, et al. Efficiency, satisfaction, and costs for remote video visits following radical prostatectomy: A randomized controlled trial. Eur Urol 2015;68(4):729–735. 25900782 10.1016/j.eururo.2015.04.002

[7] Costa MA, Yao CA, Gillenwater TJ, et al. Telemedicine in cleft care: Reliability and predictability in regional and international practice settings. J Craniofac Surg 2015;26(4):1116–1120. 26010103 10.1097/SCS.0000000000001560

[8] Hwa K, Wren SM. Telehealth follow-up in lieu of postoperative clinic visit for ambulatory surgery: Results of a Pilot Program. JAMA Surg 2013;148(9):823. 23842982 10.1001/jamasurg.2013.2672

[9] Eisenberg D, Hwa K, Wren SM. Telephone follow-up by a midlevel provider after laparoscopic inguinal hernia repair instead of face-to-face clinic visit. JSLS 2015;19(1):e2014.00205. 10.4293/JSLS.2014.00205 PMC4370039 25848178

[10] McGillicuddy JW, Gregoski MJ, Weiland AK, et al. Mobile health medication adherence and blood pressure control in renal transplant recipients: A proof-of-concept randomized controlled trial. JMIR Res Protoc 2013;2(2):e32. 24004517 10.2196/resprot.2633 PMC3786124

[11] Sathiyakumar V, Apfeld JC, Obremskey WT, et al. Prospective randomized controlled trial using telemedicine for follow-ups in an orthopedic trauma population: A pilot study. J Orthop Trauma 2015;29(3):e139–e145. 24983434 10.1097/BOT.0000000000000189

[12] Urquhart AC, Antoniotti NM, Berg RL. Telemedicine—An efficient and cost-effective approach in parathyroid surgery: Telemedicine in parathyroid surgery. Laryngoscope 2011;121(7):1422–1425. 21647908 10.1002/lary.21812

[13] Colbert GB, Venegas-Vera AV, Lerma EV. Utility of telemedicine in the COVID-19 era. Rev Cardiovasc Med 2020;21(4):583–587; doi: 10.31083/j.rcm.2020.04.188. PMID: . 33388003

[14] Chao GF, Li KY, Zhu Z, et al. Use of telehealth by surgical specialties during the COVID-19 pandemic. JAMA Surg 2021;156(7):620. 33769434 10.1001/jamasurg.2021.0979 PMC7998347

[15] Osmundsen TC, Andreassen Jaatun EA, Heggem GF, et al. Service innovation from the edges: Enhanced by telemedicine decision support. Pers Ubiquit Comput 2015;19(3):699–708.

[16] Nikolian VC, Williams AM, Jacobs BN, et al. Pilot study to evaluate the safety, feasibility, and financial implications of a postoperative telemedicine program. Ann Surg 2018;268(4):700–707. 30095477 10.1097/SLA.0000000000002931

[17] Cremades M, Ferret G, Parés D, et al. Telemedicine to follow patients in a general surgery department. A randomized controlled trial. Am J Surg 2020;219(6):882–887. 32252983 10.1016/j.amjsurg.2020.03.023

[18] Ellison EC, Pawlik TM, Way DP, et al. Ten-year reassessment of the shortage of general surgeons: Increases in graduation numbers of general surgery residents are insufficient to meet the future demand for general surgeons. Surgery 2018;164(4):726–732. 30098811 10.1016/j.surg.2018.04.042

[19] zboswell. What Is Telemedicine, Exactly?. ATA. 2020. Available from: https://www.americantelemed.org/ata-news/what-is-telemedicine-exactly [Last accessed: April 14, 2023].

